# Atypical Sotos syndrome caused by a novel splice site variant

**DOI:** 10.1038/s41439-022-00219-4

**Published:** 2022-11-16

**Authors:** Mari Minatogawa, Taichi Tsuji, Mie Inaba, Noriaki Kawakami, Seiji Mizuno, Tomoki Kosho

**Affiliations:** 1 Department of Medical Genetics, Shinshu University School of Medicine, Matsumoto, Japan; 2 Center for Medical Genetics, Shinshu University Hospital, Matsumoto, Japan; 3 Department of Orthopedics, Meijo Hospital, Nagoya, Japan; 4 Department of Orthopedics, Toyota Kosei Hospital, Toyota, Japan; 5 Department of Pediatrics, Central Hospital, Aichi Developmental Disability Center, Kasugai, Japan; 6 Department of Orthopedics, Ichinomiyanishi Hospital, Ichinomiya, Japan; 7 Division of Clinical Sequencing, Shinshu University School of Medicine, Matsumoto, Japan; 8 Research Center for Supports to Advanced Science, Shinshu University, Matsumoto, Nagano, Japan

**Keywords:** Paediatric neurological disorders, Genetic testing

## Abstract

Sotos syndrome is usually caused by haploinsufficiency of *NSD1*; it is characterized by overgrowth, craniofacial features, and learning disabilities. We describe a boy with Sotos syndrome caused by a splicing variant (c.4378+5G>A). The clinical manifestations included severe connective tissue involvement, including joint hypermobility, progressive scoliosis, pectus deformity, and skin hyperextensibility; no overgrowth was observed.

Sotos syndrome (OMIM #117550) is an autosomal dominant overgrowth syndrome with key features of early excessive growth (e.g., both height and head circumference exceed two standard deviations [SDs] above the mean), craniofacial features (e.g., high anterior hairline, frontal bossing, downslanting palpebral fissures, hypertelorism, and pointed chin), and mild to severe learning disabilities^1,2^. Other findings common to Sotos syndrome include advanced bone age, scoliosis, joint hypermobility, hearing impairment, congenital heart defects, renal anomalies, brain abnormalities, seizures, behavior problems, and an increased risk of malignant neoplasms^2^. Sotos syndrome is usually caused by haploinsufficiency of *NSD1* located on 5q35^3^. The genomic alterations responsible for Sotos syndrome are broadly classified as intragenic variants of *NSD1* and 5q35 microdeletions encompassing *NSD1*^3^. The major genomic alterations that cause Sotos syndrome differ according to ethnicity^1^. While most cases of Sotos syndrome in non-Japanese patients are caused by intragenic variants of *NSD1*, almost half of the cases in Japanese patients are caused by 5q35 microdeletions encompassing *NSD1*^1^. Although there are generally no genotype–phenotype correlations between the two genomic alterations, patients with Sotos syndrome caused by 5q35 microdeletions had more severe learning disabilities and less pronounced overgrowth than patients who exhibited intragenic variants of *NSD1*^1^. Scoliosis is also a major clinical finding, present in more than one-third of patients with Sotos syndrome^1^. Scoliosis is rarely aggravated in patients with Sotos syndrome; the presence of progressive scoliosis is associated with 5q35 microdeletions^4^. Here, we describe a patient with atypical Sotos syndrome caused by an intragenic mutation of *NSD1*, a novel splice site variant. The clinical manifestations included severe connective tissue involvement, including joint hypermobility, progressive scoliosis, pectus deformity, and mild skin hyperextensibility; no overgrowth was observed.

The proband was a 16-year-old Japanese boy who was the first child of nonconsanguineous healthy parents. Because of maternal hypertension, he was born at 40 weeks of gestation by cesarean section. At birth, his weight was 4072 g (+2.9 SD), and his length was 56 cm (+3.9 SD); occipitofrontal circumference (OFC) was not available. He showed prominent hypotonia and neonatal jaundice requiring phototherapy. At the age of 5 months, he developed pneumonia; diagnostic chest radiographs incidentally revealed scoliosis. He began walking unsupported at the age of 18 months; he began to speak single meaningful words at the age of 24 months. No abnormal karyotype was found. Although Sotos syndrome was suspected based on overgrowth at birth, global developmental delay, and facial features (e.g., broad forehead, downslanting palpebral fissures, small nose, short philtrum, and pointed chin) (Fig. 1A–D), fluorescence in situ hybridization revealed no 5q35 microdeletion encompassing *NSD1*. The proband first experienced otitis media at the age of 2 years. He developed epilepsy at the age of 3 years, which was controlled with carbamazepine. Scoliosis gradually progressed, such that he began wearing a brace at the age of 2 years (Fig. 1L); he underwent implantation of a growth-promoting vertical expandable prosthetic titanium rib at the age of 5 years (Fig. 1M, N). Subsequently, he underwent growing rod surgery; the rods were lengthened at 6-month intervals, followed by final fixation surgery at the age of 12 years (Fig. 1O, P).

**Fig. 1:**
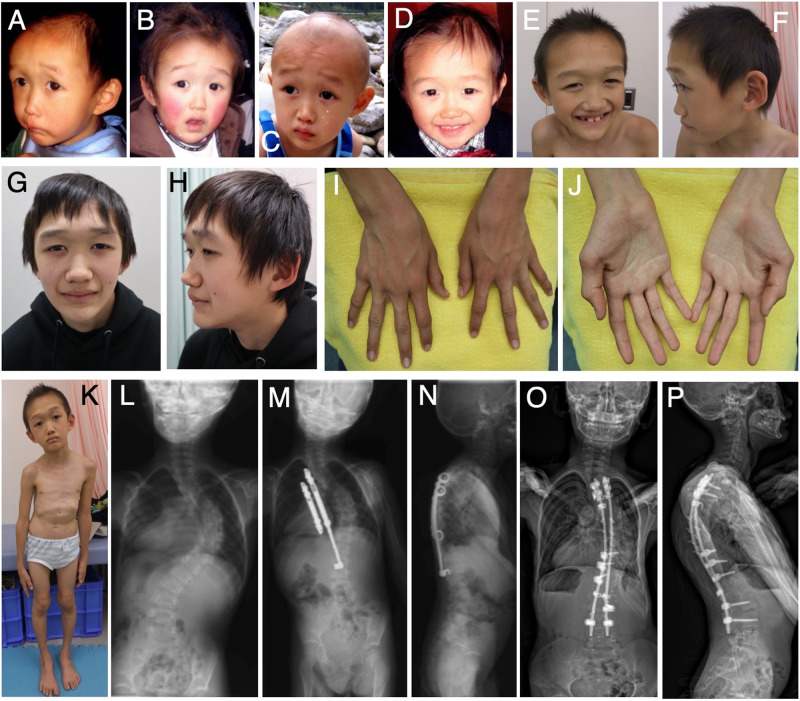
Clinical images of the proband. Facial photographs of the proband at the following ages: 1 year (**A**), 2 years (**B**), 3 years (**C**), 4 years (**D**), 8 years (**E**, **F**), and 16 years (**G**, **H**). Images of the proband’s hands at the age of 16 years (**I**, **J**). A full body image of the proband at the age of 8 years (**K**). Radiographs of the proband’s spine at the age of 2 years (**L**; before surgery), 5 years (**M**, **N**; after vertical expandable prosthetic titanium rib implantation), and 12 years (**O**; after final fixation surgery).

When the proband was 7 years of age, he underwent examination at our clinic. His main medical concerns were connective tissue-related complications, including progressive scoliosis and severe pes planus. He also showed soft and mildly hyperextensible skin; joint hypermobility; and a slender build with decreased subcutaneous fat, long and slender limbs, large hands, and large and flat feet (Fig. 1K). Consequently, a hereditary connective tissue disorder was suspected. The proband’s weight was 19.9 kg (–1.2 SD), height was 120.9 cm (–0.8 SD), OFC was 51.2 cm (–0.5 SD), and arm span was 128 cm. He showed characteristic craniofacial features (e.g., broad forehead, long and downslanting palpebral fissures, small nose, short philtrum, everted upper lip, prominent ears, high arched palate, and numerous dental caries) (Fig. 1E, F). When he was 9 years of age, array comparative genomic hybridization and gene panel analysis of hereditary connective tissue disorders (e.g., Marfan syndrome and Ehlers–Danlos syndrome) revealed no pathogenic variants.

The proband attended a special class for physically handicapped children at an elementary school because of his severe scoliosis. Evaluation by the Wechsler Intelligence Scale for Children, 4th edition, showed that he had an IQ of 61 at the age of 11 years; thus, he was admitted to a junior high school with a special class for intellectual disabilities. He had nocturia since childhood; when he was 15 years of age, urological examinations revealed bilateral vesicoureteral reflux and a reduced bladder capacity of 180 mL. Therefore, he began oral fesoterodine therapy.

We last examined the proband when he was 16 years of age; his weight was 39.1 kg (–1.6 SD), height was 158.0 cm (–1.1 SD), and OFC was 54.0 cm (–1.4 SD). He showed craniofacial characteristics (e.g., frontal bossing, downslanting palpebral fissures, and a pointed chin) (Fig. 1G, H), skeletal features (e.g., slender build, joint hypermobility in the thumbs [Beighton score 2/9], severe scoliosis, large hands with slender fingers [Fig. 1I, J], and pes planus), and skin with softness and mild hyperextensibility but without fragility or bruisability. Skin hyperextensibility was measured using the method by Remvig et al.^5^ (length of skin stretching ≥cutoff values in 4/5 areas). The systemic score in the revised Ghent criteria for Marfan syndrome^6^ was 7/20 (≧7 indicating systemic involvement), although he did not have aortic dilatation or ectopia lentis. He had neither overgrowth nor macrocephaly, suggesting an atypical form of Sotos syndrome.

An unknown disorder with connective tissue involvement was suspected; thus, whole-exome sequencing (WES) was performed. This study was approved by the Ethics Committee at Shinshu University School of Medicine. After written informed consent was obtained from the proband and his parents, genomic DNA was extracted from peripheral blood leukocytes and subjected to WES. Libraries were prepared with SureSelect Human All Exon kit V6 (Agilent Technologies, Santa Clara, CA, USA). Libraries were sequenced on NovaSeq (Illumina, San Diego, CA, USA) with 151-bp paired-end reads. After read quality was assessed using FastQC v.0.11.8 (https://github.com/s-andrews/FastQC/), the reads were aligned to the human reference genome (UCSC hg19, NCBI build 37.1) using Burrows–Wheeler Aligner v.0.7.17 (http://bio-bwa.sourceforge.net/). Read duplicates were removed using Picard v.2.20.2 (https://broadinstitute.github.io/picard/). Recalibration of base quality and variant calling were performed using Genome Analysis Tool Kit v.4.1.2.0 (https://github.com/broadinstitute/gatk/tags). Variant calling included single nucleotide variants, along with small insertions and deletions. Variants were annotated using ANNOVAR (https://doc-openbio.readthedocs.io/projects/annovar/en/latest/). Synonymous variants and intronic variants >20 bp away from exon boundaries were removed. Variants were also removed if they had minor allele frequencies (≥0.01) in our in-house clinical exome database, constructed with 367 Japanese patients; our in-house WES database, constructed with 118 Japanese patients; or public variant databases of healthy populations, including the genome Aggregation Database (gnomAD [https://gnomad.broadinstitute.org/]), the Exome Aggregation Consortium (ExAC), the Genome Medical Alliance Japan Project Whole Genome Aggregation Panel Database (GEM-J WGA, a Japanese-specific database of 7609 whole-genome sequencing variants [https://togovar.biosciencedbc.jp/doc/datasets/gem_j_wga]), and the Human Genetic Variation Database (HGVD [https://www.hgvd.genome.med.kyoto-u.ac.jp/]). Sex and paternity prediction were calculated using Peddy (https://github.com/brentp/peddy)^7^. The causative variant was confirmed by trio Sanger sequencing. WES and Sanger sequencing identified a de novo novel splice site variant of *NSD1* (NG_009821.1(NM_022455.4):c.4378+5G>A) (Fig. 2D).

**Fig. 2:**
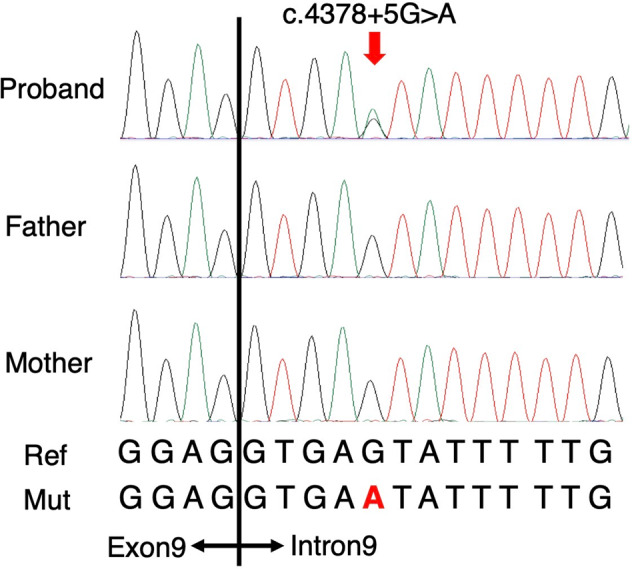
Molecular findings. Sanger sequencing electropherogram showing the de novo variant (*NSD1*:NG_009821.1(NM_022455.4):c.4378+5G>A).

The detected variant was an ultrarare novel splice site variant that was not registered in databases such as gnomAD, ExAC, GEM-J WGA, and HGVD or in our in-house databases. In silico prediction of splice site variants (http://www.fruitfly.org/seq_tools/splice.htmL) showed that the variant had a prediction score of 0.96, indicating that the variant is likely to cause splicing abnormalities and produce aberrant transcripts. Another single nucleotide substitution at the same position, c.4378+5G>C, was previously identified as a causative variant of Sotos syndrome^8^. The variant c.4378+5G>A was classified as a likely pathogenic variant according to the American College of Medical Genetics and Genomics and the Association for Molecular Pathology guidelines (PS2: de novo, PM2: absent from controls)^9^. Considering these clinical and molecular findings, the proband was diagnosed with Sotos syndrome.

Although Sotos syndrome had been suspected when the proband was an infant because of intrauterine overgrowth and craniofacial features, connective tissue involvement (e.g., skin softness and mild hyperextensibility, joint hypermobility in the thumbs, and severe scoliosis) were the main clinical manifestations thereafter. We initially presumed that he had an unknown connective tissue disorder based on his substantial connective tissue-related features along with normal OFC and nonovergrowth, which are atypical for Sotos syndrome. These atypical features might be attributed to the characteristics of the detected variant, c.4378+5G>A. However, there were no data concerning detailed clinical features in a previously described patient with Sotos syndrome who had a variant at the same position (c.4378+5G>C)^8^. The molecular consequences of this probable splicing variant might be identified through an mRNA-based analysis, but mRNA could not be obtained from the proband. Furthermore, other gene(s) might be related to connective tissue involvement in the proband, although WES excluded variants in genes relevant to known heritable connective tissue disorders.

In conclusion, we identified a novel splice site mutation of *NSD1* in a patient with atypical Sotos syndrome that included substantial connective tissue involvement without overgrowth. These observations might extend the phenotypic spectrum of Sotos syndrome.

## Data Availability

The relevant data from this Data Report are hosted at the Human Genome Variation Database at 10.6084/m9.figshare.hgv.3252.
